# Hypoxic Cell-Derived Extracellular Vesicles Aggravate Rectal Injury Following Radiotherapy *via* MiR-122-5p

**DOI:** 10.3389/fcell.2022.892575

**Published:** 2022-04-26

**Authors:** Yiqing Xu, Yulong Ge, Xuming Chen, Yingzi Zhang, Huanliang Chen, Dongli Liu, Yue Lu, Yong Liu, Wenzhi Tu

**Affiliations:** ^1^ Department of Radiation Oncology, Shanghai General Hospital, Shanghai Jiao Tong University School of Medicine, Shanghai, China; ^2^ Department of Radiotherapy, Huangpu Branch of the Ninth People’s Hospital, Shanghai Jiao Tong University School of Medicine, Shanghai, China

**Keywords:** rectal injury, hypoxia, extracellular vesicle, apoptosis, miR-122-5p

## Abstract

Radiation-induced rectal injury is a common side effect of radiotherapy. Hypoxia often occurs after radiotherapy. This study aimed to explore the bystander effect of hypoxia on radiation-induced rectal injury. *In vivo*, apoptosis increased nearby the highly hypoxic area in the rectal tissues in the mouse models of radiation-induced rectal injury, indicating the potential involvement of hypoxia. *In vitro*, flow cytometry and Western blotting showed that both hypoxia and hypoxic human intestinal epithelial crypt (HIEC) cell supernatant promoted apoptosis in normoxic HIEC cells. The pro-apoptotic effect of extracellular vesicles (EVs) derived from hypoxic HIEC cell to normoxic HIEC cells was then determined. MiR-122-5p was chosen for further studies through a microRNA (miRNA) microarray assay and apoptosis was alleviated in cells receiving miR-122-5p inhibiting hypoxic EVs. Together, our study demonstrated that the miR-122-5p containing-EVs derived from hypoxic HIEC cells promoted apoptosis in normoxic HIEC cells. Hypoxic EV-derived miR-122-5p plays a critical pathologic role in radiation-induced rectal injury and may be a potential therapeutic target.

## Introduction

Radiation therapy (RT) is a major treatment modality for patients with pelvic cancer (Nicholas et al., 2017). However, it is estimated that more than half of the pelvic cancer patients receiving RT will suffer from radiation-induced side effects; among them, radiation-induced rectal injury is a common one (Araujo et al., 2020). The clinical manifestations of radiation-induced rectal injury generally range from rectal pain, bleeding, and diarrhea (Araujo et al., 2020). Inflammation, ulcers, and hemorrhage are often observed during endoscopy. Biopsies usually reveal the presence of angiotelectasis, crypt distortion, and fibrosis (Wu et al., 2015). Presently, few strategies have been developed to treat radiation-induced rectal injury. Medications such as antioxidants and anti-inflammatory agents may assist depending on the stage of the disease (Tabaja and Sidani, 2018).

Hypoxia plays a vital role in the development of radiation-induced rectal injury (Fleckenstein et al., 2007). In addition to studies showing the involvement of hypoxia in radiation-induced lung injury (Tubin et al., 2018), our previous work showed that hypoxia also accelerates the development of radiation-induced late rectal injury by producing angiogenic cytokines (Liu et al., 2008). Through the induction of hypoxia inducible-factor-1α (HIF-1α) (Lema and Cunningham, 2010), hypoxia results in the activation of vascular endothelial growth factor (VEGF) and transforming growth factor beta pathways, which lead to inflammation and fibrosis (Ramakrishnan et al., 2014; Lei et al., 2019). Other studies have shown that nicotinamide adenine dinucleotide phosphate oxidase and reactive oxygen species contribute to hypoxic cell damage (Song et al., 2015). Moreover, clinical investigations have reported the effectiveness of hyperbaric oxygen therapy as a treatment for radiation-induced rectal injury, indicating the therapeutic role of targeting hypoxia (Oliai et al., 2012; Yoshimizu et al., 2017). However, the precise pathogenic mechanism of radiation-induced rectal injury remains unclear, and there are few studies on how hypoxia affects radiation-induced rectal injury, especially in a bystander manner.

Originated from the endosomal system or plasma membrane, EVs are a heterogeneous group of membranous particles comprising exosomes, microvesicles, and apoptotic bodies (Abels and Breakefield, 2016). Much research is undergoing on the promising role of EVs as biomarkers or intercellular messengers (Shao et al., 2018; Van Niel et al., 2018). Henrich (Henrich et al., 2020) found that EVs secret from prostate cancer promote metastasis through intercellular communication with bone marrow cells. In oral squamous cell carcinoma, it was observed that exosomal miR-1246 promotes tumor invasion by targeting the DENN/MADD domain containing 2D (Sakha et al., 2016). There is increasing evidence that the secretion of EVs and changes in their content and functions are enhanced under hypoxia (Venturella et al., 2021). In mouse models of chronic asthma, hypoxic hUCMSC-derived EVs attenuate allergic airway inflammation (Dong et al., 2021). After myocardial infarction, EV-derived miR-486-5p enhance cardiac angiogenesis *via* fibroblastic MMP19-VEGFA cleavage signaling (Li J. et al., 2021). These findings show that EVs funtions in normal tissue damage, especially under hypoxia. While molecules such as proteins, lipids, DNAs, and mRNAs are contained in EVs, miRNAs are among the most explored and best-investigated contents because of their overwhelming biological functions (Zhang et al., 2015). As mentioned above, hypoxia is involved in the pathogenic mechanism of radiation-induced rectal injury. Therefore, we hypothesized that hypoxic parts of the rectum may transfer miRNAs to the normoxic parts of the rectum through EVs, leading to the modulation of bioactivity in the latter.

## Materials and Methods

### Animal Studies

All animal study protocols were approved by the Animal Ethical Commission of the Shanghai General Hospital (No. 2020AW067). Female C57BL/6 mice (5-week-old) were purchased from Shanghai SIPPR/BK Laboratory Animal Co. Ltd. According to our previous studies, a single 25 Gy of X-ray elicited from a medical linear accelerator (Varian Clinac IX) was administered to the 1 cm-width of anal area of mice after general anesthesia. The rectal tissues of mice were collected on the 14th day after radiation following cervical dislocation for further research.

### Immunohistochemistry

The rectal tissue sections were deparaffinized and incubated in a sodium citrate buffer. Following blocking with 10% bovine serum albumin (BSA), the sections were incubated with primary antibodies against cleaved caspase-3 (casp-3) (Cell Signaling Technology, 9661, 1:100) or HIF-1α (Affinity, BF8002, 1:400) overnight at 4°C. The sections were then rinsed with phosphate buffered saline (PBS) and incubated with secondary antibodies (Jackson ImmunoResearch Inc., 111-035-003, 1:100) for 1 h at room temperature. Images of the slides were captured using a microscope (Leica, Germany). The level of hypoxia was evaluated using the immunoreactive score of HIF-1α in nuclei by two pathologists, and an average score was used for the final scores. The intensity scores were as follows, 0, negative; 1, weak; 2, moderate; and 3, strong. The frequencies of positive cells were clarified as follows, 0, negative; 1, 10% positive cells; 2, 11–50% positive cells; 3, 51–80% positive cells; and 4, more than 80% positive cells. Immunoreactive score = intensity score × frequency of positive cells (Xue et al., 2016). An immunoreactive score >8 was considered as high HIF-1α, and ≤8 was considered as low HIF-1α. The degree of cleaved casp-3 was estimated by the number of cleaved casp-3 positive cells per field.

#### Terminal Deoxynucleotidyl Transferase-Mediated dUTP Nick-End Labeling Assay

A TUNEL apoptosis kit (Roche) was used to detect apoptosis in tissue sections according to the manufacturer’s instructions. Images were captured using a microscope (Leica, Wetzlar, Germany). ImageJ software was used to estimate the percentage of TUNEL-positive areas.

#### Cell Line, Treatment and Culture

The HIEC cell line was a gift from Professor Shao (Institute of Radiation Medicine, Fudan University, Shanghai, China). The cells were cultured in RPMI-1640 medium (Wisent, Canada) containing 10% fetal bovine serum (FBS) (Wisent, Canada) and 1% penicillin–streptomycin (Gibco, United States) in a 37°C humidified incubator with an atmosphere of 5% CO_2_. For the hypoxia group, the cells were cultured under hypoxic conditions (37°C, 1% O_2_, 5% CO_2_, 94% N_2_) for 24 h. For the EV-treated group, the cells were collected for further research 48 h after the addition of EVs. The EVs were removed from FBS by ultracentrifugation (100000 × g, 20 h, 4°C) when the cultured cells were prepared for EV isolation.

### HIF-1α Immunofluorescent Detection

After rinsed with PBS, the cells were treated with 4% paraformaldehyde for 15 min followed by permeabilization with 0.1% Triton X-100 for another 15 min. Then, 1% BSA was used for block before incubation with HIF-1α primary antibody (1:500, 179483, Abcam) overnight at 4°C. The next day, the cells were incubated with Alexa Fluor 488 Goat Anti-Rabbit IgG secondary antibody (1:1000, 150077, Abcam) for 1 h in the dark, after which, the nuclei were stained with DAPI (1:1000, D9542, Sigma).

### Flow Cytometry

For the detection of apoptosis, an Annexin V-FITC Apoptosis Detection Kit (BD Pharmingen TM, United States) was used according to the manufacturer’s instructions. Briefly, the cells were digested and resuspended in binding buffer, followed by staining with Annexin V-FITC and PI in turn protected from light. They were then analyzed using an Accuri C6 Flow cytometer (BD Biosciences, United States). The total apoptosis rate was considered to be the sum of the early (lower right area of the scatter diagram) and late (upper right area of the scatter diagram) apoptosis rates.

### Isolation and Identification of EVs

To isolate EVs, 120 ml of HIEC cell culture supernatant was collected and subjected to sequential centrifugation at 300 × g for 10 min to remove cells, 2000 × g for 10 min to remove dead cells, 10000 × g for 30 min to remove cell debris, and ultracentrifugation at 100000 × g for 70 min at 4°C. The sediments were washed, resuspended in PBS, and subjected to ultracentrifugation at 100000 × g for 70 min at 4°C for final extraction (Beckman Coulter, United States).

To identify the characteristics of EVs, transmission electron microscopy (TEM) was used to observe the morphology of the extract. DiO (Beyotime, C1038, China)-labeled EVs along with DAPI staining were captured under a confocal microscope to define the cellular uptake of EVs by HIEC cells. Specific EV surface markers were examined by Western blotting.

### Western Blotting Analysis

The cells were washed with pre-cooled PBS and lysed with radioimmunoprecipitation assay buffer (Beyotime, China) containing protease and phosphatase inhibitors (Bimake, United States) when the total protein was collected. The nuclear and cytosolic proteins were extracted using a Nuclear and Cytoplasmic Protein Extraction Kit (Beyotime, P0028, China) according the manufactures’ introduction. The protein concentration was determined using a BCA Protein Quantification kit (Thermo, United States). 20 μg of total protein was electrophoresed on a sodium dodecyl sulphate-polyacrylamide gel electrophoresis gel, followed by transfer to polyvinylidene difluoride membranes (Millipore, United States). After blocking with 5% (w/v) BSA in TBST for 1 h, the membranes were incubated with primary antibodies overnight at 4°C. The next day, the primary antibodies were eliminated, and the membranes were rinsed in TBST before they were further incubated with secondary antibodies (Cell Signaling Technology, 7074, 7076, 1:3000) at room temperature for 1 h. A chemiluminescent detection system (Tanon, China) was used to visualize the blots. The primary antibodies used were as follows, HIF-1α (Abcam, 179483, 1:1000), Lamin B1 (Proteintech, 66095-1-Ig, 1:10000), *a*-Tubulin (Sigma, T8203, 1:5000), CD9 (CST, 13403, 1:1000), Alix (Santa Cruz, sc-53540, 1:500), Calnexin (Proteintech, 10427-2-AP, 1:10000), *ß*-actin (Cell Signaling Technology, 4970, 1:1000), phospho-AKT (p-AKT) (Ser473, Cell Signaling Technology, 4060, 1:1000), and γH2AX (CST, 9718, 1:1000).

### MicroRNA Inhibitor Transfection

MiR-122-5p inhibitor and negative control were synthesized by RiboBio (Guangzhou, China) and were diluted to a final concentration of 200 nM during transfection. Lipofectamine 2000 (Invitrogen, United States) was used to perform the transfection process according to the manufacturer’s instructions.

### Quantitative Real-Time PCR

cDNA was synthetized using a miRcute Plus miRNA First-Strand cDNA Kit (Tiangen, KR201, China). qRT-PCR was conducted using a miRcute Plus miRNA qPCR Kit (Tiangen, FP411, China) on a QuantStudio 6 Flex system (Life Technologies, United States) with U6 chosen to be the reference gene. The 2^−△△Ct^ method was applied for the calculation of relative expressions of genes. The primers were synthesized by RiboBio (Guangzhou, China) and were listed in Table 1.

**TABLE 1:** Primer sequences for qRT-PCR in this study.

miRNA	Primer Sequence
miR-122-5p	Forward 5′-GTG​ACA​ATG​GTG​GAA​TGT​GG-3′
	Reverse 3′-CAG​AAC​CGT​AGC​AAA​CGA​AA-5′
U6	Forward 5′-CTC​GCT​TCG​GCA​GCA​CA-3′
	Reverse 3′-TGC​GTT​TAA​GCA​CTT​CGC​AA-5′

### Microarray Assay and Bioinformatics Analysis

The miRNAs were extracted using a mirVana RNA Isolation Kit (Life Technologies, United States). The miRNA microarrays were performed using an Agilent (Santa Clara, United States) human miRNA (8 × 60 K) chip to identify differentially expressed miRNAs between hypoxic and normoxic EVs. Briefly, after quantification and integrity assessment, the total RNA sample was subjected to labeling, microarray hybridization, and washing according to the manufacturer’s protocols. Raw data were obtained using Feature Extraction software (version 10.7.1.1, Agilent Technologies). GeneSpring software (version 13.1, Agilent Technologies) was then applied to complete the standardization and subsequent management of the raw data. The differentially expressed miRNAs were screened under the criterion of a Fold change>2.0 or Fold change < −2.0, and a *p* value <0.05, and their target genes were predicted using Targetscan, microRNAorg, and PITA databases. Gene Ontology (GO) analysis and Kyoto Encyclopedia of Genes and Genomes (KEGG) analyses were performed to conduct the functional analysis of these target genes.

### Statistical Analysis

All statistical analysis were performed using GraphPad Prism 7 software (La Jolla, United States). Student’s t-test was applied for statistical analysis between two groups. A minimum of three biological replicates were performed, and a value of *p* < 0.05 was considered statistically significant. The data were showed as the mean ± standard deviation.

## Results

### Rectal Tissue Apoptosis is Aggravated Near Hypoxic Area in Mouse After Radiation

**FIGURE 1:**
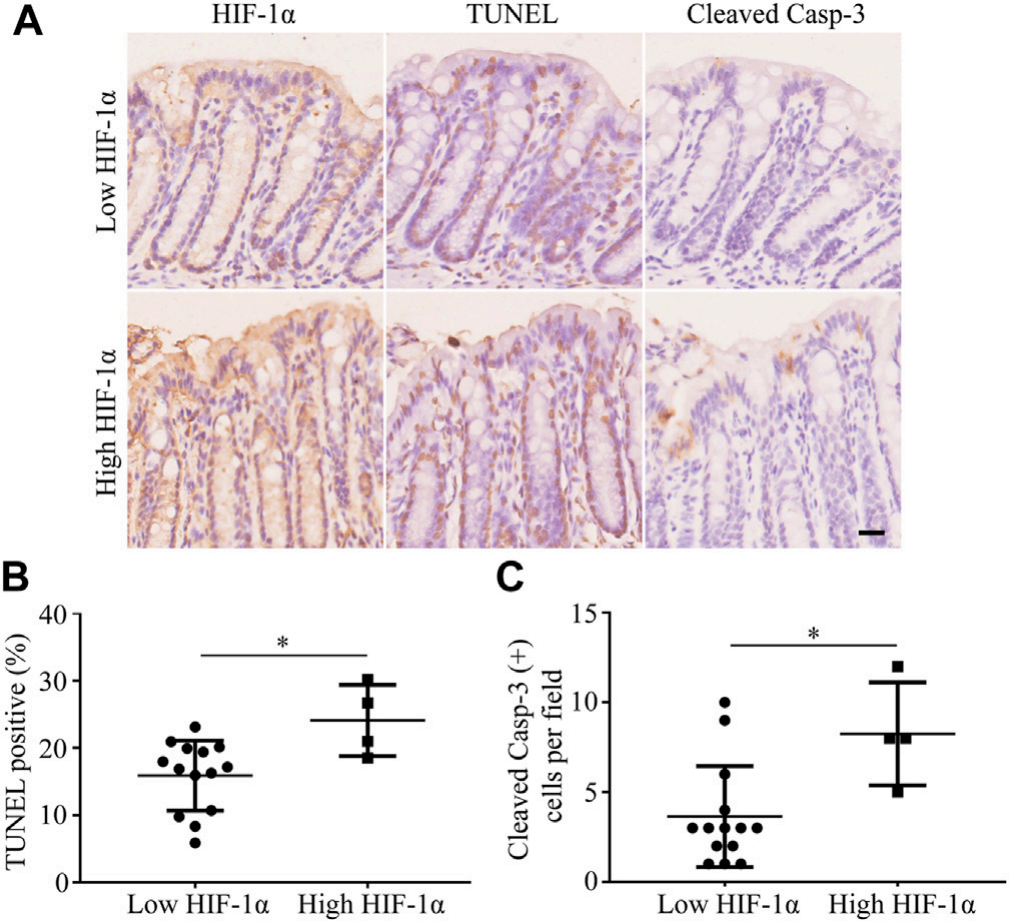
Apoptosis is increased adjacent to high-HIF-1α area in rectum of mouse after 25 Gy radiation. **(A)** Representative pictures of TUNEL and cleaved casp-3 staining in low-HIF-1α and high-HIF-1α groups *in vivo*. Scale bar: 20 μm. **(B)** The extent of TUNEL and **(C)** the counts of cleaved casp-3 positive cells per field were positively correlated with the degree of hypoxia. *p < 0.05.

In the mouse models of radiation-induced rectal injury with a 25 Gy radiation, adjacent sections of the rectum were separately stained for HIF-1α, TUNEL, and cleaved casp-3, and the degree of each staining was measured as mentioned above. The sections were then divided into high-HIF-1α and low-HIF-1α groups based on their immunoreactive scores for HIF-1α. As shown in Figure 1A, both the extent of TUNEL staining (Figure 1B) and the counts of cleaved casp-3 positive cells per field (Figure 1C) were positively correlated with the expression of HIF-1α in their adjacent sections, indicating that hypoxia may promote injury to surrounding tissues after radiation.

### Hypoxia and Hypoxic Supernatant Increase the Apoptosis of Normoxic HIEC Cells

**FIGURE 2:**
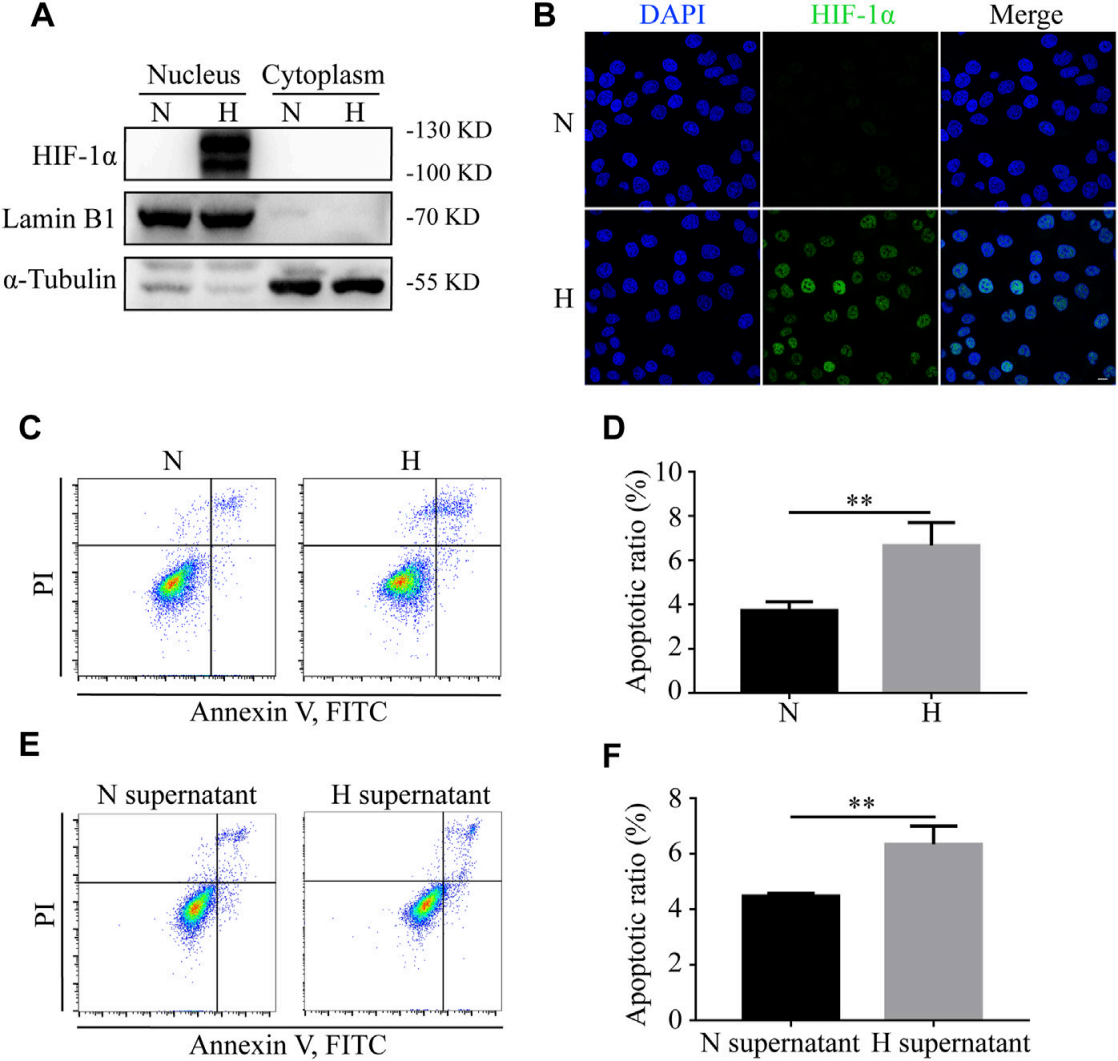
Hypoxia and hypoxic supernatant promote apoptosis in normoxic HIEC cells. **(A)** Western blotting and **(B)** IF detection of the levels of HIF-1α in nuclear and cytoplasmic fractions of HIEC cells under normoxia or hypoxia. **(C, D)** Flow cytometry investigation of apoptosis of HIEC cells under normoxia or hypoxia. **(E, F)** Flow cytometry investigation of apoptosis of HIEC cells cultured in normoxic or hypoxic HIEC cell supernatant. Scale bar: 10 μm. N, normoxia; H, hypoxia; N supernatant, normoxic supernatant; H supernatant, hypoxic supernatant. **p < 0.01, ***p < 0.001.

To determine the remote effects of hypoxia on normoxic tissues, HIEC cells were used *in vitro*. We first cultivated HIEC cells in either normoxic or hypoxic conditions for 24 h. Western blotting showed that the hypoxic sensor HIF-1α had a higher expression in the nuclear fraction under hypoxic conditions compared to the normoxic group (Figure 2A). IF detection also exhibited the translocation of HIF-1α in the nucleus under hypoxia (Figure 2B), indicating that the hypoxic culture conditions were successfully established. The Annexin V-FITC staining assay demonstrated that the apoptosis rate of HIEC cells increased under hypoxic conditions (Figures 2C,D). To further clarify the biological influence of hypoxia, the supernatant of hypoxic HIEC cells was collected and utilized to cultivate normoxic cells. After 48 h of incubation, the pro-apoptotic effect of the hypoxic cell supernatant was confirmed by flow cytometry (Figures 2E,F). Collectively, these data indicate that both hypoxia and hypoxic cell supernatants promote apoptosis in normoxic HIEC cells.

### Hypoxic HIEC Cell-Derived EVs Promote Apoptosis in Normoxic HIEC Cells

**FIGURE 3:**
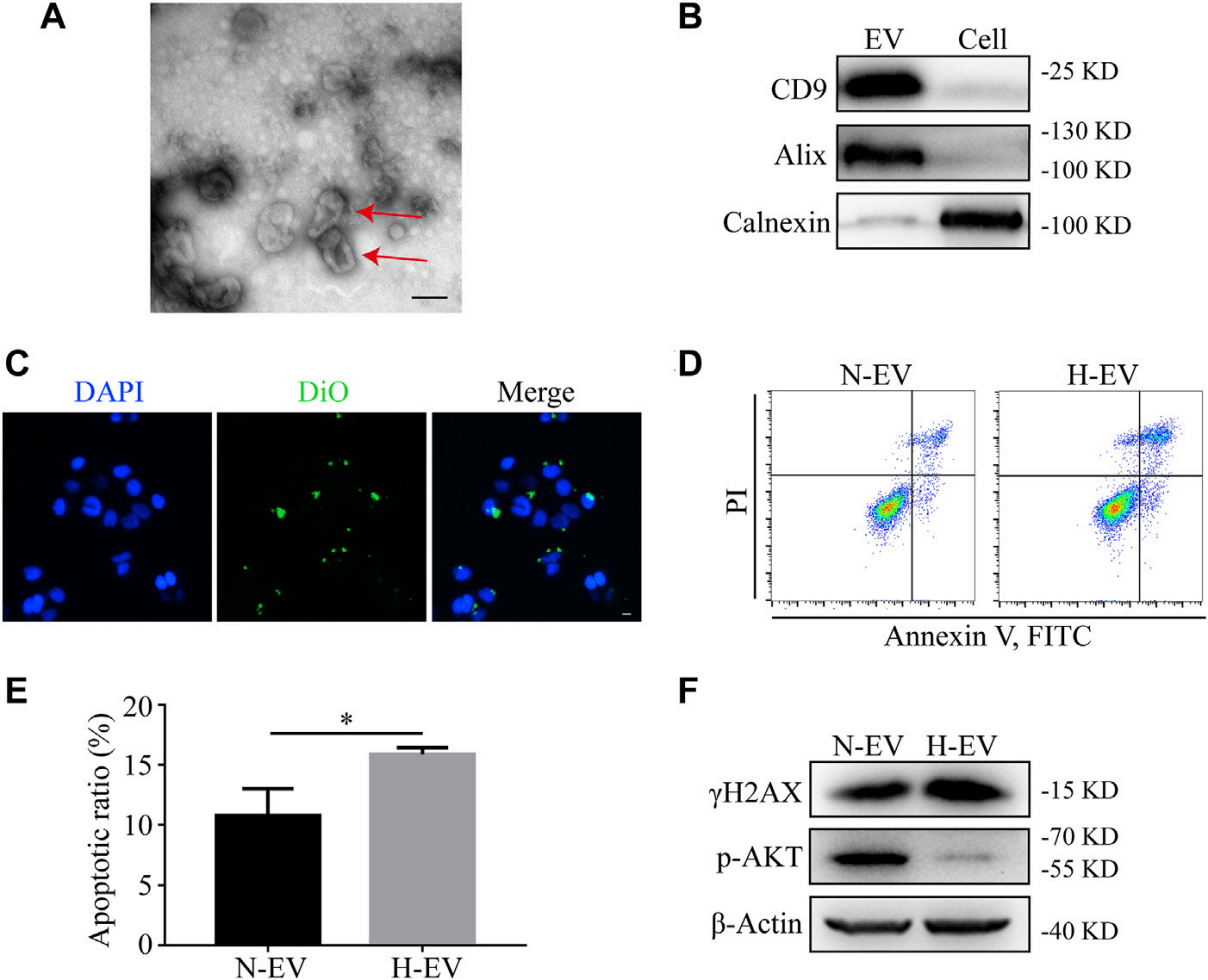
Hypoxic EVs enhance apoptosis in normoxic HIEC cells. **(A)** Representative TEM image of the saucer-like structures of EVs (red arrow). Scale bar: 100 nm. **(B)** Western blotting analyzed the EV markers. **(C)** Representative confocal images showing the uptake of EVs. Scale bar: 10 μm. **(D,E)** Flow cytometry investigation of apoptosis and **(F)** Western blotting analysis of the expression of γH2AX and p-AKT in HIEC cells given either normoxic or hypoxic EVs. *ß*-Actin was used as the internal control. N-EV, normoxic EVs; H-EV, hypoxic EVs. *p < 0.05.

To investigate whether EVs were involved in hypoxia-induced apoptosis, EVs from either normoxic or hypoxic HIEC cells were extracted by ultracentrifugation. As shown by TEM, typical saucer-like structures with a diameter of 50–200 nm were captured (Figure 3A). The levels of EV markers CD9 and Alix along with the endoplasmic reticulum marker Calnexin were detected by Western blotting (Figure 3B). The fluorescent confocal images also showed the uptake of DiO-labeled hypoxic HIEC cell-derived EVs by normoxic HIEC cells (Figure 3C). After confirmation of successful EV extraction, we added normoxic or hypoxic EVs into the medium of normoxic HIEC cells. After 48 h, Annexin V-FITC staining revealed that hypoxic EVs promoted apoptosis in HIEC cells compared to normoxic EVs (Figures 3D,E). In addition, we further evaluated the expression of apoptosis-related DNA damage marker γH2AX and the apoptosis-related AKT pathway. Western blotting showed that the expression of γH2AX increased while that of p-AKT decreased owing to the addition of hypoxic EVs (Figure 3F).

### Microarray Assay of Differentially Expressed MiRNAs Between Normoxic EVs and Hypoxic EVs

**FIGURE 4:**
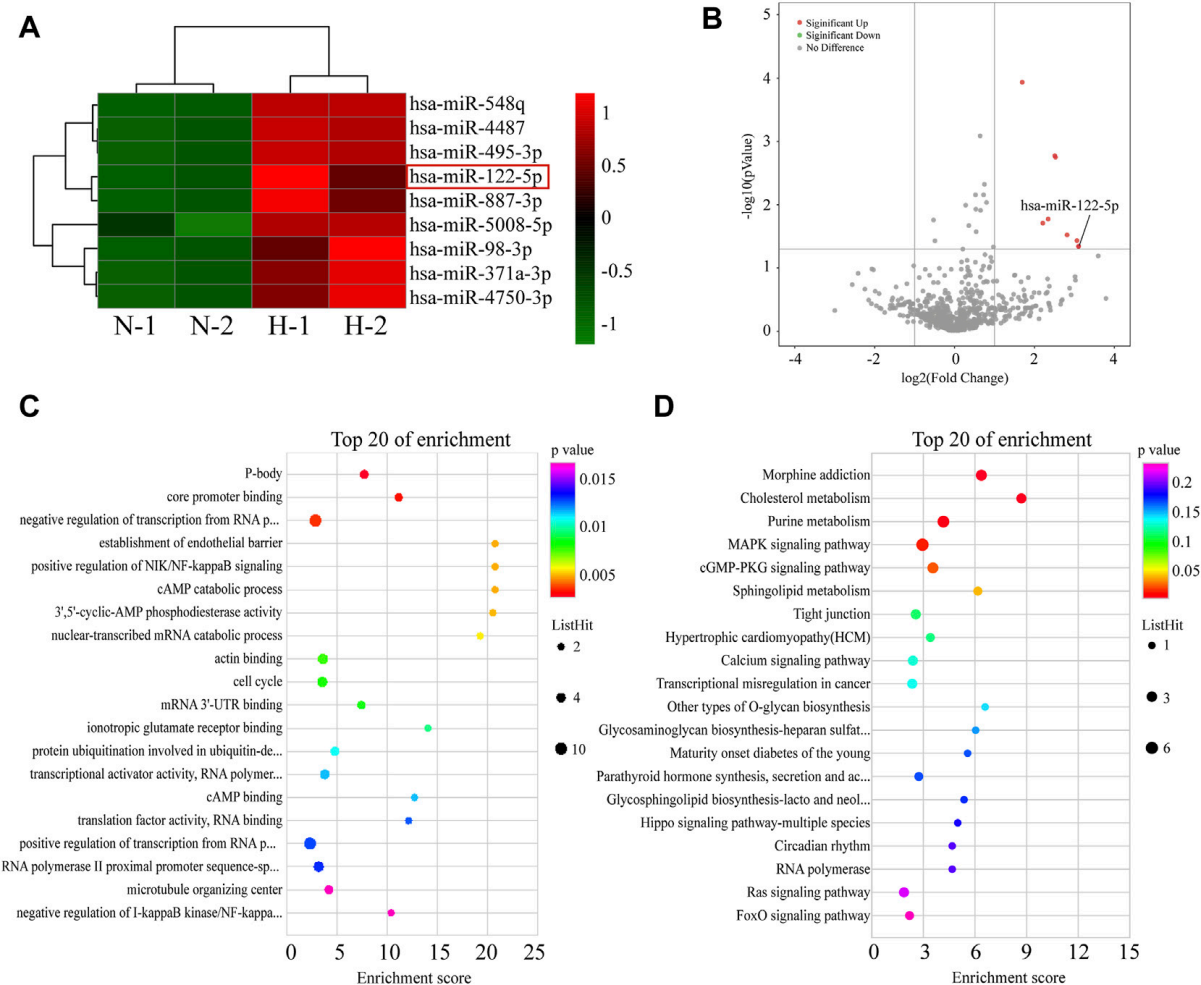
Screening of differentially expressed miRNAs between EVs from normoxic or hypoxic HIEC cells. **(A)** The differential miRNA clustering showed in heat map and **(B)** volcano plot. The top 20 terms in **(C,D)** GO and KEGG analysis of targeted genes of differential miRNAs. N, normoxic EV-derived miRNAs; H, hypoxic EV-derived miRNAs.

It can be concluded from above that hypoxic HIEC cell-derived EVs promoted injury of normoxic HIEC cells. To further elucidate the molecular mechanism responsible for this, a miRNA microarray assay was conducted. The differential miRNA clustering heat map (Figure 4A) and a volcano plot (Figure 4B) demonstrated that there were nine miRNAs upregulated in hypoxic EVs compared to normoxic EVs (Fold change>2.0 or Fold change < −2.0, and *p* < 0.05). Then, GO and KEGG analyses were performed to analyze the functions of 136 predicted target genes of these differentially expressed miRNAs. The top-ranked terms are displayed in the bubble charts. Apoptosis related pathways, for example, NF-kappaB and MAPK signaling, were among them (Figures 4C,D).

### MiR-122-5p is Involved in the Hypoxia-Induced Bystander Effects of EVs in Normoxic HIEC Cells

**FIGURE 5:**
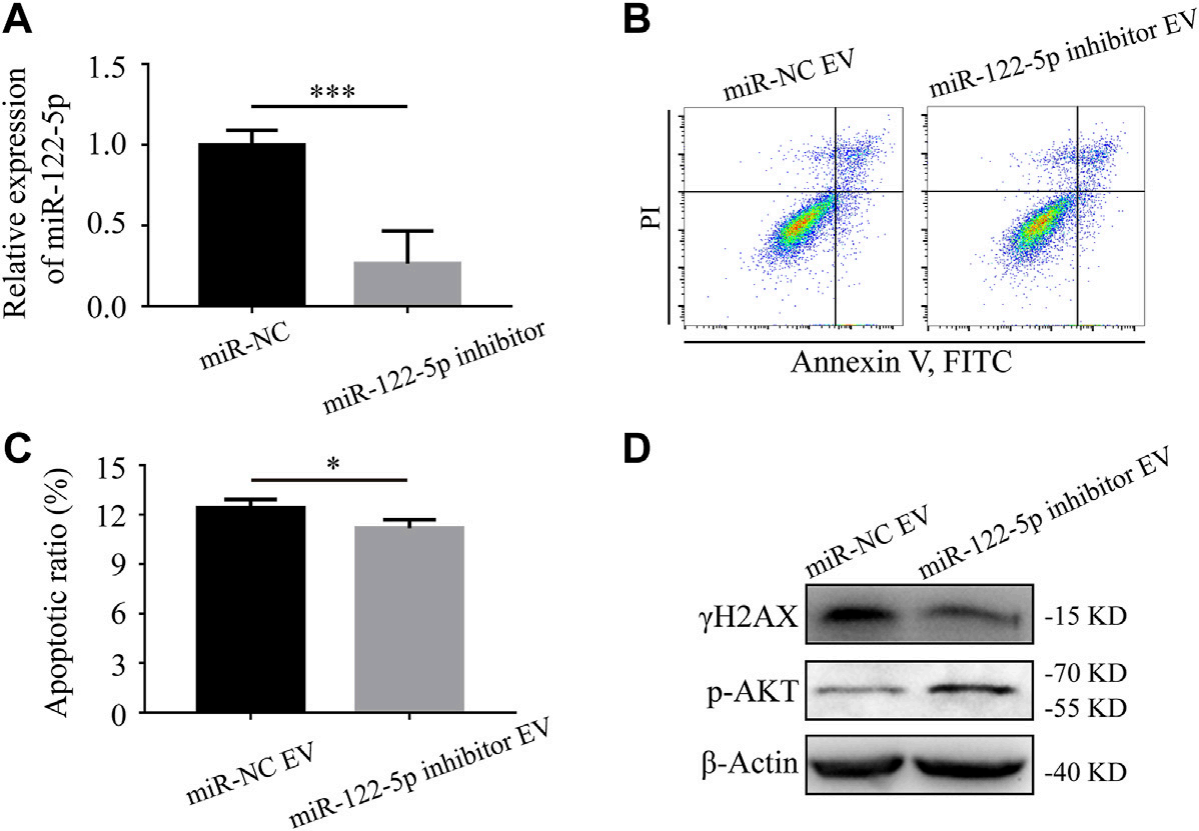
Hypoxic EV-derived miR-122-5p mediates the apoptosis of normoxic HIEC cells. **(A)** The transfection efficiency of miR-122-5p after 24 h of transfection with miR-122-5p inhibitor. U6 was used as the endogenous control. **(B, C)** Flow cytometry investigation of apoptosis and **(D)** Western blotting analysis of the expression of γH2AX and p-AKT of HIEC cells given the hypoxic EVs from either miR-122-5p inhibitor group or negative control group. *ß*-Actin was used as the internal control. NC, negative control. **p < 0.01, ***p < 0.001.

**FIGURE 6:**
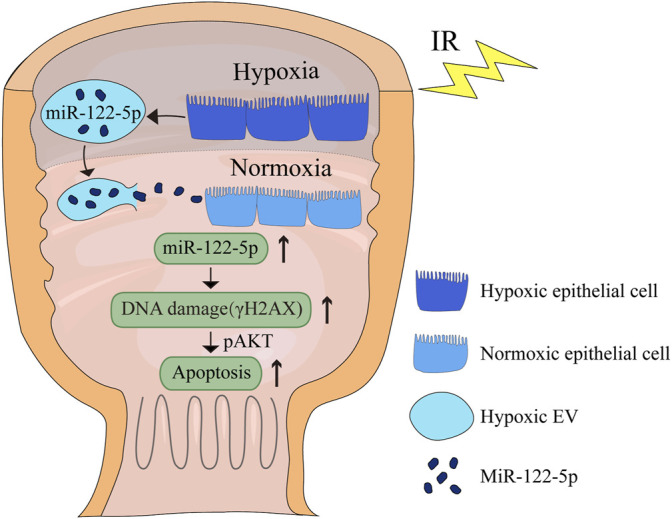
Schematic diagram of the effect of hypoxic EV-derived miR-122-5p on normoxic rectum. IR, ionizing radiation.

Based on the microarray assay, miR-122-5p was selected for further research because of its highest fold change among all the upregulated miRNAs (Table 2). To determine the role of miR-122-5p in hypoxic EVs-aggravated apoptosis, HIEC cells were transfected with either miR-122-5p inhibitor or negative control and cultured under hypoxic conditions. qRT-PCR showed that miR-122-5p decreased at 24 h after transfection (Figure 5A). EVs from hypoxic HIEC cells at 24 h after transfection were extracted and used to culture normoxic HIEC cells as previously described. Annexin V-FITC staining showed that the apoptosis rate in HIEC cells treated with hypoxic miR-122-5p inhibiting EVs was significantly lower than that in the control group (Figures 5B,C). In addition, Western blotting also showed an increased expression of p-AKT along with a decreased expression of γH2AX upon treatment with hypoxic miR-122-5p inhibiting EVs compared to the control groups (Figure 5D). These results indicated that EV-derived miR-122-5p secreted by hypoxic HIEC cells promoted apoptosis in normoxic HIEC cells, and perhaps the AKT pathway was involved in this process (Figure 6).

**TABLE 2:** Significantly differentially expressed EV-derived miRNAs under hypoxia for 24 h in HIEC cells.

Hypoxia versus Normoxia	Fold Change	*p* Value
Upregulated		
hsa-miR-122-5p	8.604761	0.045137
hsa-miR-98-3p	8.588404	0.045548
hsa-miR-5008-5p	8.350174	0.036899
hsa-miR-887-3p	7.036182	0.029985
hsa-miR-495-3p	5.760533	0.001771
hsa-miR-4487	5.686698	0.001685
hsa-miR-371a-3p	5.06989	0.016827
hsa-miR-4750-3p	4.620697	0.019536
hsa-miR-548q	3.233005	1.16E-04

## Discussion

As mentioned above, radiation-induced rectal injury not only causes great physical and mental pain in oncology patients receiving pelvic radiotherapy, but also limits the clinical application of radiotherapy. Tissue hypoxia is one of the features of radiotherapy (Rabbani et al., 2010). In this study, we observed aggravated apoptosis adjacent to highly hypoxic regions in the mouse rectum after radiation, which suggested that hypoxia may promote injury to the surrounding area. Then, the apoptosis rates were found to increase in hypoxic HIEC cells, and in normoxic HIEC cells cultured in supernatant from hypoxic HIEC cells. After extraction and identification, hypoxic HIEC cell-derived EVs were added to normoxic HIEC cells, which were found to induce apoptosis and γH2AX expression. miR-122-5p was chosen for further study through miRNA microarray assays. Compared to negative controls, HIEC cells receiving miR-122-5p inhibiting hypoxic EVs had a lower apoptosis rate, decreased γH2AX expression, and increased p-AKT expression.

Vascular and epithelial abnormalities caused by radiation contribute to a hypoxic microenvironment through the upregulation of HIF-1α (Vujaskovic et al., 1998; Lu et al., 2012). There is growing evidence that HIF activation leads to the abnormal blood supply, mucosal barrier injury, inflammation, and immune dysregulation in the gut under hypoxic conditions induced by radiation or ischemic reperfusion (Khanna et al., 2019; Singhal and Shah, 2020), indicating the important role of hypoxia in gastrointestinal diseases. Similar results were also found in this study when aggravated apoptosis was observed in tissues adjacent to the high-HIF-1a area in the mouse rectum after radiation. Thus, in addition to the direct effects mentioned above, we assumed that hypoxia may have a wider impact on tissue damage in an indirect way. Recently, hypoxia has been found to take part in radiation-induced bystander effects (RIBE), which refer to effects in non-irradiated cells responding to signals released from irradiated cells (Zhang et al., 2021). RIBE results from multiple mechanisms including oxidative DNA damage (Havaki et al., 2015), epigenetic factors (Aypar et al., 2011), oxidative metabolism (Azzam et al., 2003), and cytokine release (Facoetti et al., 2006), increasing the risk of secondary carcinogenesis and normal tissue injury (Wang et al., 2015). Several studies have shown that exposure to hypoxia leads to an increased release of signaling factors from irradiated cancer cells, contributing to damage in non-irradiated bystander cells (Zhang et al., 2021). In the present study, we found that hypoxic supernatants significantly promoted apoptosis in HIEC cells under normoxic conditions. This allowed us to reminisce how hypoxic HIEC cells transmitted injury signals to normoxic HIEC cells, accounting for an expanded injury area, and how hypoxia acted in the injury cascade after radiation through its crosstalk with components in the hypoxic microenvironment as a potential cause of distant damage.

As potential biomarkers or drug delivery in multiple diseases, EVs are found to be important in cell communications, including RIBE (Du et al., 2020). Research reports that EVs isolated from the bone marrow of total-body irradiated mice mediate radiation-induced immune and inflammatory responses in non-irradiated mice (Szatmari et al., 2019). Radiation-induced miR-34c enriched-EVs cause oxidative stress in non-irradiated HaCaT cells (Rastogi et al., 2018). Intestinal epithelial cells are also capable of secreting exosome-like vesicles (Van Niel et al., 2001). Jiang (Jiang et al., 2016) found that EVs extracted from intestinal tissues participate in regulating the intestinal tract immune functions. On top of that, EVs secreted by gut microbiota also exert a significant impact on gut immunomodulation and barrier integrity (Chelakkot et al., 2018; Liu et al., 2021). In the present study, we discovered the pro-apoptotic effects of EVs secreted from hypoxic HIEC cells on normoxic HIEC cells, again highlighting the important biological role of EVs in gut disorders.

EVs, along with their containing miRNAs are attracting growing attention because of their potent biological effects under various conditions (Zhang et al., 2015). Serum miR-122-5p expression can be used as a potential biomarker for gastric and renal cancers (Heinemann et al., 2018; Zhang et al., 2019). In normal tissues, however, miR-122-5p also functions. In myocardial injury, miR-122-5p aggravates oxidative stress (Song et al., 2021). Inflammatory cytokines are reduced in lipopolysaccharide-induced lung injury when miR-122-5p is inhibited (Li Q. et al., 2021). In our previous study (Ge et al., 2020), we found that miR-122-5p promoted radiation-induced rectal injury through inhibiting cell cycle and apoptosis regulator 1. In this study, miR-122-5p was identified as one of the upregulated EV-derived miRNAs in hypoxic HIEC cells compared to normoxic cells. Upon knockdown of miR-122-5p with miRNA inhibitors, miR-122-5p was found to be involved in the pro-apoptotic effect of EVs on normoxic HIEC cells from hypoxic cells. These results, along with our previous studies, together suggest the important role of miR-122-5p in promoting intestinal injury, not only induced by radiation, but also by hypoxia.

Studies have shown that hypoxia accumulates DNA double-strand break (γH2AX) formation and promotes apoptosis (Kumareswaran et al., 2012; Wozny et al., 2020). In the present study, the level of γH2AX was measured to evaluate if there was the effect of DNA damage brought by hypoxic EVs to HIEC cells which perhaps contributed to the increased apoptotic rate. The increased γH2AX observed in cells receiving hypoxic EVs indicated exacerbated DNA damage caused by hypoxic EVs. The expression of γH2AX declined when given miR-122-5p inhibiting EVs, which suggested that EV-derived miR-122-5p functioned in this process. Moreover, the Akt (Ser473) signaling pathway regulates a wide range of cellular functions, including cell survival and apoptosis (Zhang et al., 2018). Studies have shown that upregulation of p-AKT inhibits apoptosis in normal tissue damage induced by hypoxia (Cai et al., 2018; Wei et al., 2020). In agreement with this, we found that the expression of p-AKT was negatively correlated with the apoptosis rate when given EVs extracted from different treatments, suggesting the involvement of p-AKT in the pro-apoptotic effect of hypoxic EV-derived miR-122-5p.

Few studies have been conducted on the influence of pathological hypoxic guts on their normal counterparts. In summary, in this study, we found that hypoxic EV-derived miR-122-5p aggravated apoptosis in normoxic HIEC cells involving γH2AX and p-AKT. The current study may enrich the research on the pathogenic mechanisms of radiation-induced rectal injury. Researchers have reported the application of antagomiRNAs as remedies (Zhou et al., 2021). Our study provides a rationale for the treatment of radiation-induced rectal injury. Future studies on the specific mechanisms of miR-122-5p are warranted.

## Data Availability

The raw data supporting the conclusion of this article will be made available by the authors, without undue reservation.
